# Editorial: Novel technologies in the diagnosis and management of sleep-disordered breathing, volume III

**DOI:** 10.3389/frsle.2025.1767653

**Published:** 2026-01-22

**Authors:** Ding Zou, Henri Korkalainen

**Affiliations:** 1 Center for Sleep and Vigilance Disorders, Institute of Medicine, Sahlgrenska Academy, University of Gothenburg, Gothenburg, Sweden; 2 Department of Technical Physics, University of Eastern Finland, Kuopio, Finland; 3 Diagnostic Imaging Center, Kuopio University Hospital, Kuopio, Finland

**Keywords:** artificial intelligence, comorbidity, KPAP, obstructive sleep apnea, patient-centered care, pediatric, telemedicine, wearable

Advances in artificial intelligence (AI) have accelerated dramatically in recent years, exemplified by the release of increasingly capable models such as Gemini 3.0, Nano Banana Pro, Opus 4.5, and ChatGPT 5.2 in late 2025. As technological progress continues at an unprecedented speed, the contrast with progress in healthcare sector is striking (Nguyen et al., 2025). Despite substantial scientific advances, the clinical pathways of sleep-disordered breathing (SDB) and obstructive sleep apnea (OSA) have remained largely anchored to traditional paradigms even as the sleep community has repeatedly called for reform (Korkalainen et al., 2024). Change is inherently challenging, yet there comes a tipping point where decisive action is required, much like the paradigm shift executed by the Canadian Cardiovascular Society and the Canadian Heart Failure Society in redefining heart failure classifications (Virani et al., 2025). The 17 articles featured in Volume III highlight how innovative diagnostics, deeper pathophysiological understanding, therapeutic breakthroughs, and integrated care models are converging to reshape management of SDB. Together, they signal a growing momentum toward a more precise, accessible, and patient-centered model of SDB care. This editorial synthesizes these contributions and reflects on how the field can leverage this momentum to advance a new era of personalized sleep medicine.

## Expanding the diagnostic toolkit

Accurate and accessible diagnosis is the foundation of effective SDB management, yet current methods often fall short. The traditional reliance on in-laboratory polysomnography (PSG) has created diagnostic bottlenecks and failed to capture the longitudinal variability of SDB. To overcome these challenges, several contributions in this Research Topic illustrate how the diagnostic toolkit for SDB is being expanded through technological innovation.

The push for home-based monitoring is evident in the evaluation of novel form factors. Gell et al. demonstrate that a simple finger-worn ring pulse oximeter can approximate PSG-derived metrics, such as oxygen desaturation index and hypoxic burden, with a promising ability to detect moderate-to-severe OSA. The results highlight the potential of streamlined sensors to expand access to longitudinal OSA assessment and enable more responsive, data-driven care models. Similarly, Zhang et al. explore the utility of ballistocardiography (BCG), a contactless piezoelectric sensor stripe placed under the patient, for OSA severity assessment. The promising results illustrate the potential for “install-and-forget” home monitoring systems that do not require patient compliance with wearables. While the integration of wearable and contactless sensors into clinical pathways will require further validation in diverse populations and clarity on appropriate use cases, the simpler measurement setups have the potential to revolutionize long-term monitoring and accessible care.

Moving deeper into physiological signals, Yanney et al. revisit Pulse Transit Time (PTT), specifically the respiratory swing (PTTrs), as a diagnostic tool. PTT refers to the interval required for a pulse wave to travel from the heart to a peripheral site (e.g., finger) and was used for arousal event detection (Pitson et al., 1994). In a large observational study of children, they found that PTTrs may offer a practical, non-invasive complement to oximetry to improve OSA detection in children, potentially offering a scalable alternative to PSG in resource-limited secondary care settings. By demonstrating a connection between PTTrs and OSA severity, the work supports interest in physiologically grounded metrics that can enhance diagnostic transparency and interpretability, an important contrast to machine-learning–based approaches often criticized as “black box” methods. Furthermore, Sarkez-Knudsen et al. present an ambitious study protocol for a cutting-edge approach to measuring the elusive symptom of “sleepiness” (Heffron et al., 2025). By utilizing ultra-long-term subcutaneous electroencephalography (EEG) combined with ecological momentary assessment both before and during treatment, they aim to objectively quantify sleep pressure in the patient's natural environment, moving beyond the subjective recall bias of questionnaires. This work exemplifies a broader shift toward longitudinal, patient-centered monitoring strategies that may ultimately refine how residual symptoms and treatment responses are quantified.

Despite technological developments in diagnostic measurements, clinical physical examination remains vital. Guimarães et al. highlight the “scalloped tongue” as a simple, accessible clinical sign. Their study found that this diagnostic sign, particularly with a neck circumference ≥40 cm, is a strong predictor of severe OSA, suggesting that a quick oral exam can significantly refine risk stratification in primary care. On the imaging front, Lun et al. demonstrate that cervical tracheal ultrasonography can reliably capture dynamic airway movement and dimensional changes in OSA patients, revealing greater caudal tracheal displacement and larger tracheal displacement than healthy controls. This adds a biomechanical dimension to our understanding of airway collapsibility and offers a new potential screening modality. Finally, the importance of cultural adaptation in screening tools is underscored by Oboleviciene and Miseviciene, who successfully validated the Lithuanian version of the Pediatric Sleep Questionnaire (PSQ) (Chervin et al., 2000), ensuring that diagnostic tools are accessible across linguistic barriers.

## Comorbidities and pathophysiology

Several papers in this Research Topic elucidate the bidirectional relationships between sleep apnea and systemic disease. Lu et al. provide compelling evidence of the link between OSA and diabetic complications, even after accounting for metabolic and lifestyle confounders. In a study of 228 subjects with Type 2 Diabetes (T2D), they found that those with moderate-to-severe OSA had significantly higher odds of developing diabetic peripheral neuropathy (DPN) (adjusted odds ratio 2.2) and showed significant correlations with small fiber damage as measured by corneal confocal microscopy. This underscores the need to view OSA as a metabolic stressor that potentially accelerates neurodegeneration. Conversely, treating metabolic disease may improve sleep. In an observational study, Wilhelmsen-Langeland et al. show that a concentrated, micro-choice-based bio-behavioral treatment for T2D was associated with sustained reductions in insomnia symptoms, daytime sleepiness, and questionnaire-based OSA risk over 12 months. This suggests that empowering patients to manage metabolic health through lifestyle micro-choices may benefit multiple aspects of sleep health. At the molecular level, Wei et al. review the role of the NLRP3 inflammasome, identifying it as a key mediator linking intermittent hypoxia to cardiovascular and neurocognitive complications. This mechanistic insight identifies potential targets for future pharmacological interventions, such as Glucagon-Like Peptide-1 (GLP-1) receptor agonists, that could inhibit the inflammasome and block the downstream consequences of sleep apnea even when the obstruction itself is not fully resolved (Malhotra et al., 2024). Finally, Du et al. analyze the large U.S. Nationwide Inpatient Sample to evaluate the impact of OSA on COVID-19 outcomes. They confirmed that patients with OSA had higher risks of respiratory failure and heart failure. They also reported a paradoxical finding, reduced in-hospital mortality among older male patients with OSA. While the authors suggested possible explanations, including “ischemic preconditioning” (Sanchez-de-la-Torre et al., 2018) or protective effects from continuous positive airway pressure (CPAP), the possibility of survival bias inherent in a hospitalized cohort could not be excluded.

## Innovations in therapy

Messineo et al. provide a comprehensive reassessment of why decades of technological refinements in positive airway pressure (PAP) therapy have yielded only modest, if any, gains in long-term adherence, highlighting that many long-standing assumptions about airway mechanics may be physiologically misguided. They reframe the physiology of upper-airway collapse under PAP therapy and propose that rather than occurring primarily during inspiration, airway narrowing often initiates at end-expiration, when pharyngeal cross-sectional area, lung volume and tracheal traction are lowest (Morrell et al., 1998). This challenges decades of device design focused on increasing inspiratory pressure and relieving expiratory pressure, strategies that have not meaningfully improved long-term adherence, and may have possibly increased Treatment Emergent Central Sleep Apnea (TECSA) occurrence rate (Johnson and Johnson, 2005).

Two companion studies translate this physiological insight into clinical practice. V-Com^®^, a flow-dependent inline resistor, selectively reduces inspiratory PAP but preserves expiratory PAP, and resolved residual TECSA in a small group of patients (Noah et al., 2025). Kairos positive airway pressure (KPAP) advances this approach by decreasing inspiratory pressure and delivering full therapeutic pressure only near end-expiration (White et al., 2024). In two randomized studies, KPAP had similar effect to CPAP on the residual apnea hypopnea index (AHI), while reducing leak by nearly 50%. Additionally, at pressure levels of 9 and 13 cmH_2_O, KPAP was preferred by 69% and 84% of patients, respectively (White et al., 2024). These studies collectively support the notion that timing of exposure to pressure may be the key to comfort and physiologic stability. If validated in broader populations, such approaches may become a novel PAP innovation since the introduction of bilevel positive airway pressure (BiPAP) (Sanders and Kern, 1990).

For those who cannot tolerate PAP, pharmacological alternatives are emerging (Lisik and Zou, 2025a). Larramona Carrera reports three illustrative pediatric cases in which combined atomoxetine and oxybutynin therapy produced substantial reductions in OSA severity when adenotonsillectomy and CPAP had failed, with two patients achieving >50% improvement in AHI after 1 month of treatment. These early observations suggest a potential role for medication-based therapy in complex pediatric OSA and underscore the need for controlled pediatric trials to define efficacy, safety, and target phenotypes for pharmacologic OSA treatment.

## Telemedicine, AI, and integrated models

The final theme in this topic addresses how the care pathway for SDB is being transformed by telemedicine and AI, enabling more integrated and proactive management models. As the volume of patients with OSA continues to grow worldwide, health systems face mounting pressure to provide timely diagnosis and longitudinal care despite limited specialist resources. Here, novel care models harnessing digital health technology and AI-driven decision support are crucial. Zou et al. provide a forward-looking perspective on redefining telemedicine AI. They argue that AI is not just a tool for automation but a mechanism to enable “treatable traits” based management (Lisik and Zou, 2025b). By integrating multimodal data, from comorbidities to lifestyle factors, AI can facilitate a seamless, personalized care loop from screening to long-term adherence. This theoretical framework is supported by a practical feasibility study by Hediger-Parolini et al., who demonstrated the feasibility of continuously monitoring blood pressure, sleep–wake patterns, physical activity, and CPAP usage using a combination of consumer-grade wearables and a centralized data platform. Despite technical challenges in data synchronization that must be overcome to realize the full potential of these ecosystems, the study shows that multi-sensor remote monitoring is both acceptable to patients and capable of producing integrated longitudinal datasets.

The superiority of cohesive care models over fragmented fee-for-service models is demonstrated by two studies in this Research Topic. Salinas et al. report that patients enrolled in a clinically integrated, comprehensive sleep-care program reported significantly higher satisfaction with access to care, diagnostic processes, and ongoing support compared with those receiving traditional care. Importantly, this enhanced satisfaction appears to translate into more favorable outcomes across the patient care trajectory. Riney et al. study real-world, long-term evidence from a similar comprehensive care pathway, reporting PAP adherence rates of 82.6% at 1 year, and 74.2% of patients at 2 years, significantly higher than traditional models. These findings may directly translate into reduced burden for the healthcare system (Malhotra et al., 2025) and underscore the need for investments in coordinated, patient-centered infrastructure to enhance the effectiveness of disease management.

## Conclusion

Decades of research have made it clear that OSA management can and should be improved. The real challenge now lies in integration. How do we synthesize data from a ring oximeter, a subcutaneous EEG, and a tracheal ultrasound into a coherent clinical picture? How do we implement the AI algorithms proposed by Zou et al. without losing the humanistic elements emphasized by Salinas et al.? The answers lie in the continued interdisciplinary collaboration demonstrated in this Research Topic. As we refine these technologies and models, we move closer to a future where sleep apnea is detected earlier, managed easier, and treated not just as a disorder of breathing, but as a cornerstone of holistic health.
